# A Study on Pharmacokinetic Functionalities and Safety Margins of an Optimized Simvastatin Nanoformulation

**DOI:** 10.3390/ph16030380

**Published:** 2023-03-01

**Authors:** Aftab Ahmad, Unnikrishnan Meenakshi Dhanalekshmi, Kailasam Koumaravelu, Arul Prakash Francis, Shah Alam Khan, Mohammed F. Abuzinadah, Nandakumar Selvasudha

**Affiliations:** 1 Health Information Technology Department, Faculty of Applied Studies, King Abdulaziz University, Jeddah 21589, Saudi Arabia; 2 Pharmacovigilance and Medication Safety Unit, Center of Research Excellence for Drug Research and Pharmaceutical Industries, King Abdulaziz University, Jeddah 21589, Saudi Arabia; 3 College of Pharmacy, National University of Science and Technology, Muscat PC 130, Oman; 4 Centre of Molecular Medicine and Diagnostics (COMMAND), Saveetha Dental College and Hospitals, Saveetha Institute of Medical & Technical Sciences, Saveetha University, Chennai 600077, India; 5 School of Pharmacy, PRIST University, Manamai 603127, India; 6 Department of Medical Laboratory Technology, Faculty of Applied Medical Sciences, King Abdulaziz University, Jeddah 21589, Saudi Arabia; 7 Department of Biotechnology, Pondicherry University, Puducherry 605014, India

**Keywords:** nanoformulation, simvastatin, chitosan, PPAR-γ, SREBP-2.0

## Abstract

A pharmaceutical formulation with favorable pharmacokinetic parameters is more likely to be efficacious and safe to overcome the failures of the drug resulting from lack of efficacy, poor bioavailability, and toxicity. In this view, we aimed to evaluate the pharmacokinetic functionalities and safety margin of an optimized CS-SS nanoformulation (F40) by in vitro/in vivo methods. The everted sac technique was used to evaluate the improved absorption of a simvastatin formulation. In vitro protein binding in bovine serum and mice plasma was performed. The formulation’s liver and intestinal CYP3A4 activity and metabolic pathways were investigated by the qRT-PCR technique. The excretion of cholesterol and bile acids was measured to demonstrate the formulation’s cholesterol depletion effect. Safety margins were determined by histopathology as well as fiber typing studies. In vitro protein binding results revealed the existence of a high percentage of free drugs (22.31 ± 3.1%, 18.20 ± 1.9%, and 16.9 ± 2.2%, respectively) compared to the standard formulation. The controlled metabolism in the liver was demonstrated from CYP3A4 activity. The formulation showed enhanced PK parameters in rabbits such as a lower C_max_, clearance, and a higher T_max_, AUC, V_d_, and t_1/2_. qRT-PCR screening further proved the different metabolic pathways followed by simvastatin (SREBP-2) and chitosan (PPAR-γ pathway) in the formulation. The results from qRT-PCR and histopathology confirmed the toxicity level. Hence, this pharmacokinetic profile of the nanoformulation proved it has a unique synergistic hypolipidemic modality.

## 1. Introduction

Comprehensive knowledge of in vitro and in vivo characteristics is vital in developing novel pharmaceuticals. This study describes various steps tangled for polymeric nanoparticles to reach systemic circulation after oral administration. Nanoparticles that permeate the gut wall can enter the systemic circulation and be distributed to target organs if they do not bind to plasma proteins. Still, plasma protein binding is vital for other pharmacokinetic parameters [1]. Hence, it would be beneficial to understand the in vitro parameters of binding and permeability, which could provide information on the in vivo biodistribution of polymeric nanoparticles. Numerous research studies have been explored in the past to establish a correlation between in vitro and in vivo methods. During the drug discovery and development process, data generation from Absorption, Distribution, Metabolism, and Excretion (ADME) has considerably advanced through automated technology platforms combined with high-throughput liquid chromatography–mass spectrometry (LC/MS/MS) bioanalysis. Assessment of passive permeability, P-gp substrate, metabolic stability, liver microsomes, and whole hepatocyte models are the commonly methods used for in vitro ADME studies. In vivo pharmacokinetic (PK) data, including drug clearance (Cl), bioavailability (F%), exposure (AUC), half-life (t_1/2_), and distribution volume (L), are acquired using animal models [2].

It Is prudent to gain further understanding of in vitro/in vivo correlation and PK/PD reports at an early stage. Some marketed drugs approved by the FDA such as atorvastatin and simvastatin, with an absolute bioavailability of 14% and 5%, respectively [2,3], have become the highest selling drugs despite their poor PK profiles. Interestingly, more than 30% of marketed drugs have a relatively low bioavailability (F < 10%), almost 50% of the drugs have moderate-to-high clearance, while 17% show a high clearance rate [4]. Hence, considering only PK data to screen compounds might exclude some potential drug candidates such as atorvastatin, which could not have become developed further based on its PK profile. PD reports could be facilitated by rodent and a non-rodent toxicology studies of at least a 14-day duration and ADME/PK safety pharmacology studies. Simvastatin, initially made available in 1988, is a well-known hydroxy-methylglutaryl co-enzyme A (HMG-CoA) reductase inhibitor. At a maximum dosage of 80 mg per day, it results in an average decrease of 47% in LDL cholesterol (LDL-C), along with decreases in extremely LDL cholesterol, triglycerides, and apolipoprotein B, and a slight increase in HDL cholesterol [5]. Simvastatin has few undesirable properties in terms of solubility, Log D, in vitro microsomal instability, and permeability/efflux data, with high in vivo clearance and low bioavailability [6].

Hence, this research study aimed to focus on third-generation controlled drug delivery systems based on rectifying both formulation and biological barriers. In this study, nanotechnology and a pH-sensitive smart polymer were utilized to overcome the formulation barriers, which in turn were proposed to overcome the biological barriers for better pharmacological activities. Therefore, the optimized nanoformulation F40 (simvastatin-loaded chitosan nanoparticles, which were previously optimized) is hypothesized to have improved physicochemical properties than conventional drugs and current research mainly focused on evaluating this optimized nanoformulation F40 in terms of its pharmacokinetic profile (ADME/PK) and its safety margins.

## 2. Results

### 2.1. In Vitro Absorption

The experiment performed by an everted intestinal sac method showed that absorption of simvastatin from formulation F40 was markedly elevated as compared to standard simvastatin as shown in Table 1. Although absorption of standard simvastatin was initially high, it was decreased till the end of the experiment. However, formulation F40 showed decreased absorption in mucosae from 27 ± 0.90 to 16 ± 0.41 μg/mL after 75 min, indicating increased intestinal absorption. This is because simvastatin has a low in vivo availability not only due to its poor solubility and first-pass metabolism but also its inhibition of absorption by efflux transporters such as P-gp in the intestine, which was markedly decreased by the excipients added in formulation F40, thereby enhancing intestinal passage and permeability of simvastatin, increasing its bioavailability.

### 2.2. In Vitro Plasma Protein Binding (Distribution)

The results of the BSA binding test showed that at a given concentration, the average protein binding of nanoformulation F40 was 77.02 ± 4.58% and that of pure simvastatin was 95.00 ± 3.1%. In addition, pure simvastatin resulted in a higher percentage of protein binding compared to nanoformulation F40 coated with chitosan. The free drug present in the supernatant of nanoformulation F40 was found to be 22.31 ± 3.1%, which was higher than pure simvastatin. In the experiment involving human plasma, the free drug available for nanoformulation F40 was found to be 18.20 ± 1.9% (for pure simvastatin 4.3 ± 1.0%), whereas, for mice plasma, it was found to be 16.9 ± 2.2% for formulation F40 and for pure simvastatin 4.0 ± 0.7%. As shown in Table 2, there are variations in the results of plasma as compared to the BSA test and the percentage of binding was less. It was observed that the free drug available in mice plasma was slightly less than in human plasma which might be due to the presence of plasma esterase in mice plasma that hydrolyzes the simvastatin released from formulation F40, whereas the absence of this enzyme in human plasma renders more free drug availability. However, deviation in the results was less which might be due to the controlled release of simvastatin from formulation F40 as well as due to differences in species. Therefore, there might be differences in the percentage of free drugs and other PK parameters, which should be further confirmed by clinical studies. Nevertheless, our studies showed that the percentage of the free drug for formulation F40 was higher as compared to standard simvastatin, which indicates that it can produce a beneficial biodistribution effect.

### 2.3. Metabolism

#### 2.3.1. CYP3A4 Activity of Standard Simvastatin and Formulation F40

The mRNA levels of CYP3A4 in the liver and intestines were measured using qRT-PCR to investigate whether there was any alteration in the pharmacokinetics of simvastatin in formulation F40. This was assumed by observing the modified metabolism of F40-treated mice in the liver as simvastatin is primarily eliminated in the liver via metabolism. Results showed that, as compared to the standard simvastatin-treated group, the formulation F40-treated group had less CYP3A4 expression in both the intestine and liver. Metabolism via the intestine was less as the expression of CYP3A4 mRNA was minor in the intestine as compared to the liver. The decreased expression of hepatic CYP3A4 mRNA (threefold) in the formulation F40-treated group as compared to the standard simvastatin group (fold expression—7.90) might be due to the size as it is a nanoformulation and formation of intermolecular bonding for the presence of novel excipients due to a controlled release of simvastatin.

#### 2.3.2. Metabolic Pathway of Standard Simvastatin and the Formulation

**Figure 1:**
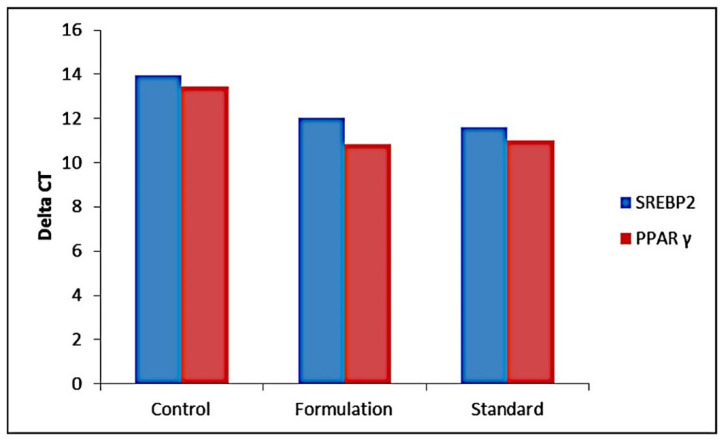
Gene expression study of the standard and CS-SS nanoformulation for transcriptional factors SREBP-2 and PPAR-γ.

This study showed that fold expression for SREBP-2 was higher for standard simvastatin (5.02) than formulation F40 and transcription factor PPAR-γ fold expression (6.06) was greater for formulation F40 than for standard simvastatin as shown in Figure 1, indicating an increased lipid metabolism through this pathway due to the presence of chitosan. This also indicates that both simvastatin and chitosan follow different metabolic pathways, which could avoid possible toxicity. Simvastatin also expresses PPAR-γ due to the anti-inflammatory effect exerted by it and indirectly induces the activity of PPAR-γ.

### 2.4. Excretion

#### 2.4.1. Food Intake and Body Weight

Animals in all groups were healthy and active for up to ten weeks of study. At the end of this study, some of the group II animals were inactive and group III animals were observed to have some behavioral changes, which were suggested due to certain muscular dystrophy produced by standard simvastatin. All groups gained weight during the experimental period except the formulation F40-treated group as depicted in Table 3. There was a minor difference in food consumption among experimental groups. Therefore, any differences among groups in the present study can be attributed to the fiber effect.

#### 2.4.2. Fecal Dry Weight

As shown in Table 3, during this study, the fecal dry weight was not significantly altered in groups I, II, and III. In contrast, the total fecal dry weight excreted by animals in the formulation F40-treated group was higher after treatment and increased from 0.32 ± 0.002 (8 weeks) to 0.49 ± 0.005 g/day (16 weeks).

#### 2.4.3. Neutral Sterol and Bile Acids

This study showed that only trace amounts of total cholesterol and bile acid were excreted in feces of control, high-fat diet, and standard simvastatin-treated animals during the whole experimental period, whereas certain amounts of fecal total cholesterol and bile acid were found in feces of formulation F40-treated animals.

#### 2.4.4. HPTLC for Individual Bile Acid and Sterols

**Figure 2:**
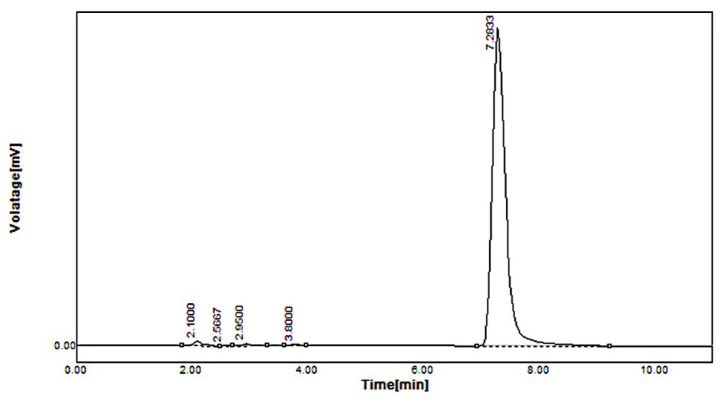
HPLC of standard simvastatin spiked in plasma.

**Figure 3:**
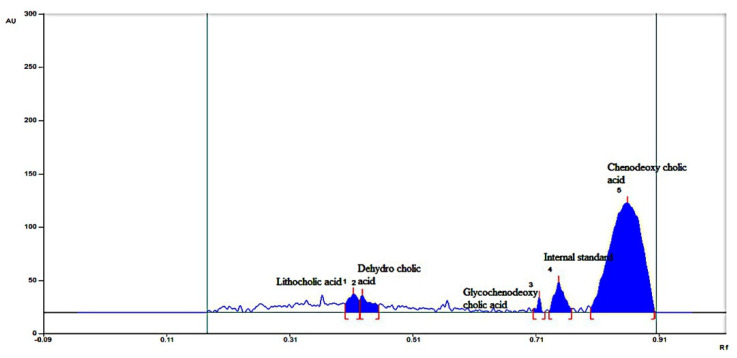
HPTLC analysis of bile acids present in feces of CS-SS nanofomulation-treated mice.

**Figure 4:**
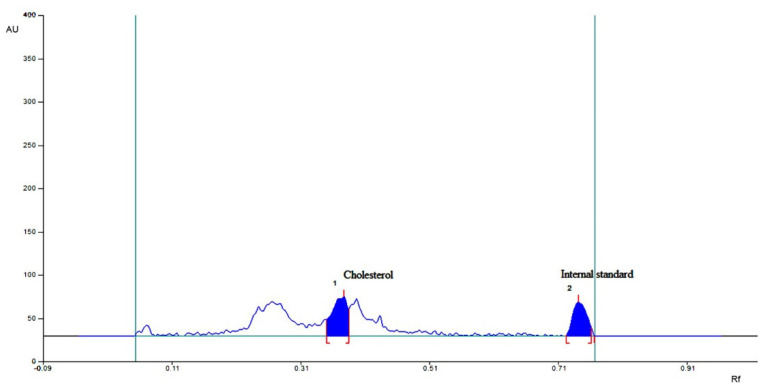
HPTLC analysis of sterols present in feces of CS-SS nanoformulation-treated mice.

HPTLC revealed that cholesterol excretion in feces increased from 1.71 ± 0.28 to 2.89 ± 0.19 mg/day/animal in the formulation F40-treated group compared to other groups. These results were compared using HPLC peaks. The bile acid chenodeoxycholic acid increased from 0.19 ± 0.001 to 4.02 ± 0.21 mg/day/animal of fecal matter in the formulation F40 group compared to the control group as shown in Figure 2, Figure 3 and Figure 4. The lithocholic acid (a secondary metabolite of chenodeoxycholic acid) and the ratio of secondary to primary metabolite decreased from 5.89 ± 0.84 to 3.78 ± 0.61 mg/day/animal and from 4.67 ± 0.42 to 2.42 ± 0.18, respectively, in the formulation F40 group. The major metabolic products of cholesterol, viz. corprostanol and cholestanol, were not significantly increased and peaks were not seen in the densitogram for groups IV as shown in Figure 4. This indicates that chitosan in formulation F40 reduced the conversion of cholesterol and primary bile acids.

**Figure 5:**
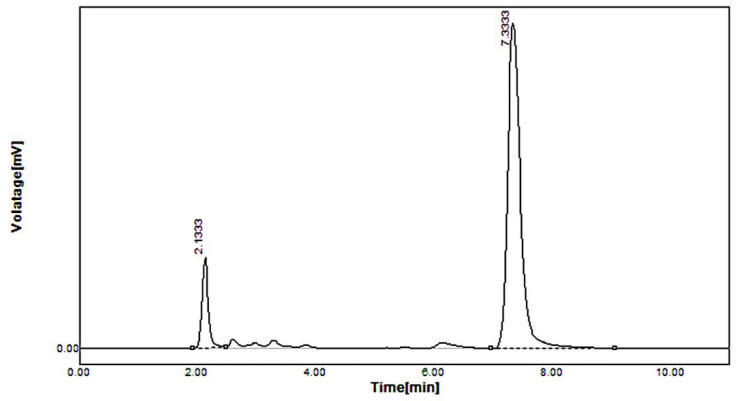
HPLC of simvastatin and its metabolite in feces of standard simvastatin-treated mice.

**Figure 6:**
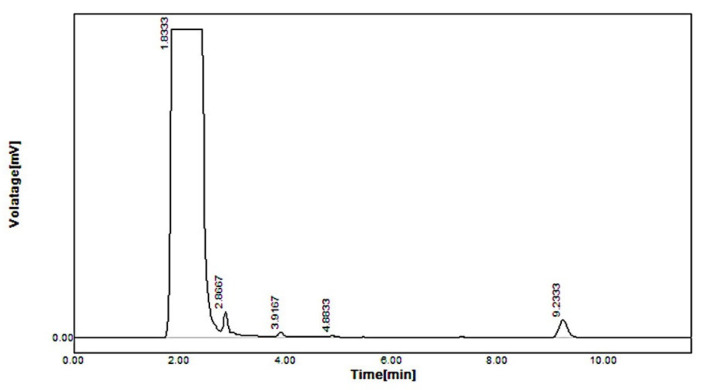
HPLC of simvastatin and its metabolite in feces of CS-SS nanoformulation-treated mice.

The concentrations of simvastatin/simvastatin metabolite (18.98 ± 0.20 ng/mL/23.12 ± 1.3 ng/mL) were found to be higher in standard simvastatin-treated mice feces, as shown in Figure 5, whereas, in the formulation F40-treated group, the concentrations were very low. Simvastatin was not detected and simvastatin metabolite was found in traces as depicted in Figure 6. The reduced concentration of simvastatin and its metabolite in feces is due to the encapsulation of simvastatin in the polymer shell of nanoformulation F40, which inhibits its exposure to gut metabolism, rendering a controlled-release pattern. In contrast, standard simvastatin is involved in gut wall metabolism and acid degradation, causing more excretion in feces, thereby leading to low bioavailability.

### 2.5. In Vivo Pharmacokinetic Study

The pharmacokinetic study conducted in rabbits was used to quantify SS and its active metabolite SSA. The mean plasma concentration profiles of SS and SSA as a function of time obtained after a 10 mg oral dose of both standard and test are shown in Table 4. Further, all the pharmacokinetic parameters of nanoformulation F40 were determined by software (Kinetica 5.0, Thermo Fisher Scientific, Waltham, MA, USA). Due to extravascular administration, non-compartmental analysis has been opted.

**Figure 7:**
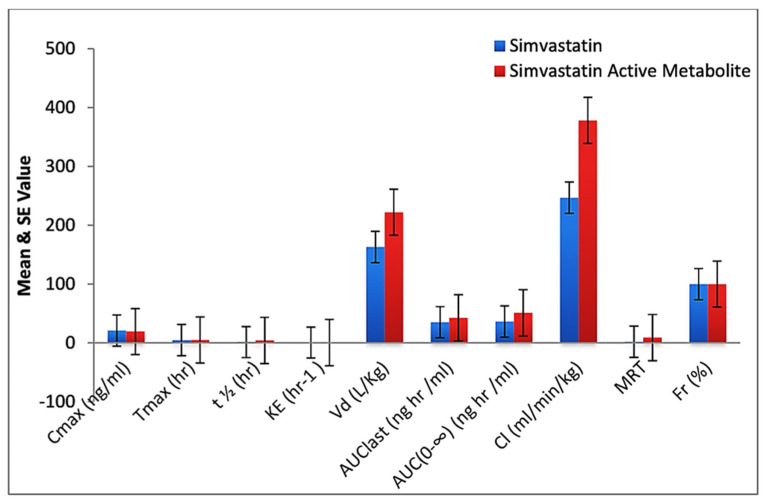
Comparison of pharmacokinetic parameters of simvastatin and simvastatin active metabolite in plasma of standard-treated rabbit.

**Figure 8:**
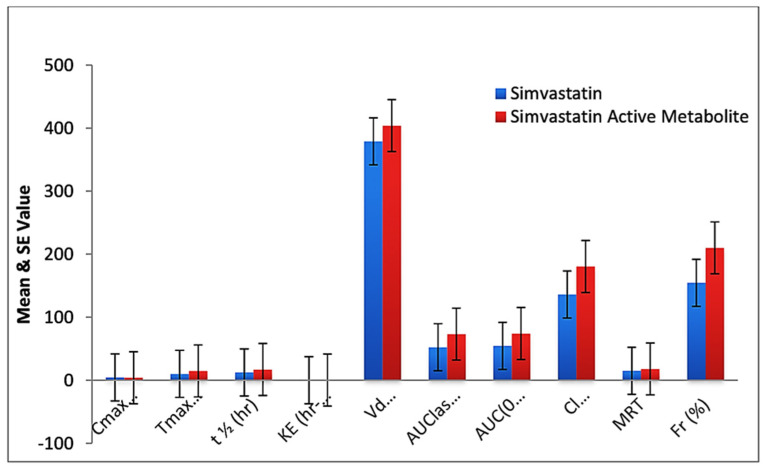
Comparison of pharmacokinetic parameters of simvastatin and simvastatin active metabolite in plasma of CS-SS nanoformulation-treated rabbit.

Lower plasma levels of simvastatin and simvastatin acid were observed with formulation F40 than with standard drug after oral administration. The AUC of formulation F40 (53.17 ng/mL, 73.11 ng/mL) and the T_max_ F40 (10 h, 14.56 h) were significantly higher than for standard simvastatin (36.38 ng/mL, 51.11 ng/mL and 4.72 h, 5 h). The C_max_ was lower for formulation F40 (4.33 ng/mL, 3.98 ng/mL) than for standard simvastatin (21.12 ng/mL, 19.42 ng/mL). The higher T_max_, AUC, and a lower C_max_ indicate the sustained-release properties of formulation F40. Generally, the drug molecules are absorbed rapidly from GIT due to an improved dissolution rate by a reduced particle size, an increased surface area, and diffusional layer thickness (nanoparticle formation and intermolecular hydrogen bonding). The mucoadhesion and controlled delivery of the drug from formulation F40 were responsible for sustained release, leading to a low C_max_ but a prolonged T_max_ and AUC. Half-life and MRT were also higher for nanoformulation F40 than for standard drug (Figure 7). A higher V_d_ (378.90 ± 112.3 ng/mL, 404.00 ± 134.98 ng/mL) and K_E_ and a lower Cl (135.78 ng/mL, 180.34 ng/mL) for nanoformulation F40 (Figure 8) than for standard simvastatin also confirmed the sustained-release property and lower plasma protein binding. In the case of standard simvastatin, a lower C_max_ and Cl, and a higher T_max_, AUC, V_d_, t_1/2_, K_E_, and MRT were observed, showing a steady-state concentration. *p* values were found to be significant for the C_max_, T_max_, t_1/2_, K_E_, V_d_, AUC last AUC(0–∞), Cl, and MRT at *p* < 0.05. Relative bioavailability or bioequivalence is the most common measure for comparing the bioavailability of one formulation of the same drug to another. The mean responses such as the C_max_ and the AUC were considered to determine relative bioavailability.

The AUC refers to the extent of bioavailability, while the C_max_ refers to the rate of bioavailability. The relative bioavailability of formulation F40 was 154%, and 209% compared to that of the pure drug simvastatin. There was only a little increase in the bioavailability of formulation F40. As simvastatin is a narrow therapeutic-indexed drug, an increase in drug concentration in the plasma might enhance toxicity. When the results of PD studies were compared with PK studies, a reduction in the TC level and an increase in relative bioavailability in the case of nanoformulation F40 were observed. Thus, the results obtained in the PK study are well supported by the PD studies, which showed the same hypolipidemic activity of nanoformulation F40 compared with standard drugs with reduced doses and negligible toxicity.

### 2.6. Histopathology

#### 2.6.1. Observation

**Figure 9:**
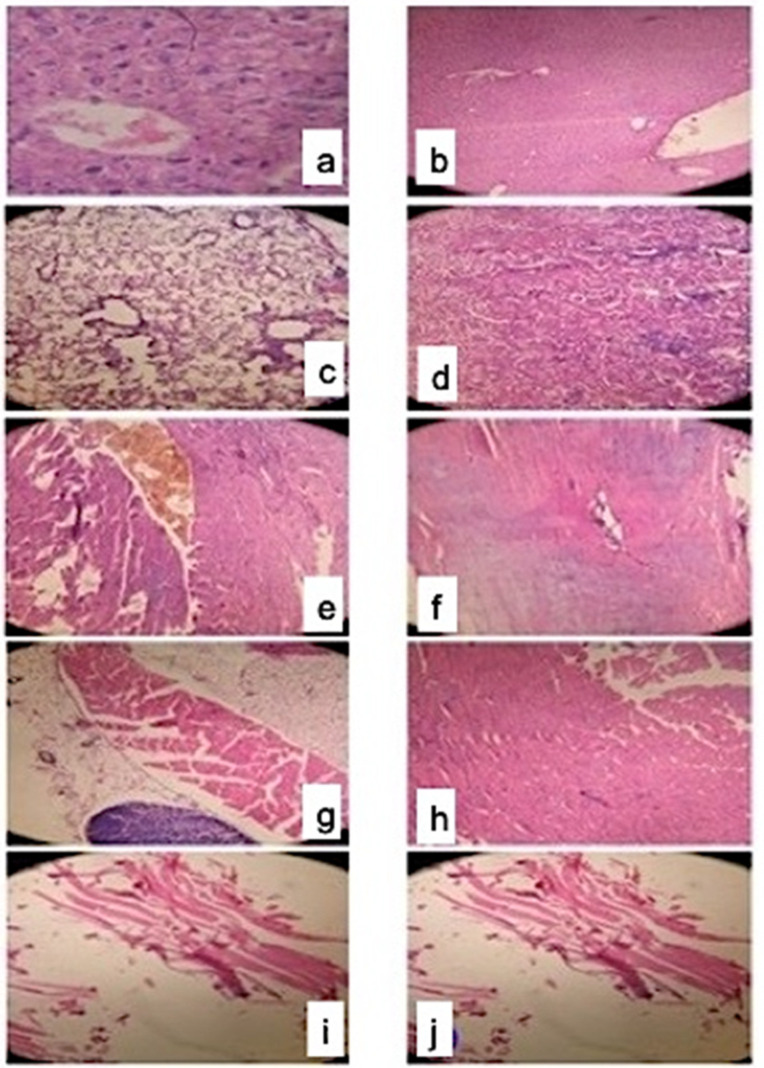
Histopathological evaluation of vital organs and muscles of group I mice (n = 3). (**a**—liver, **b**—brain, **c**—lung, **d**—kidney, **e**—heart, **f**—vastus medialis, **g**—biceps brachii, **h**—biceps femoris, **i**—soleus, and **j**—tibialis cranialis.).

**Figure 10:**
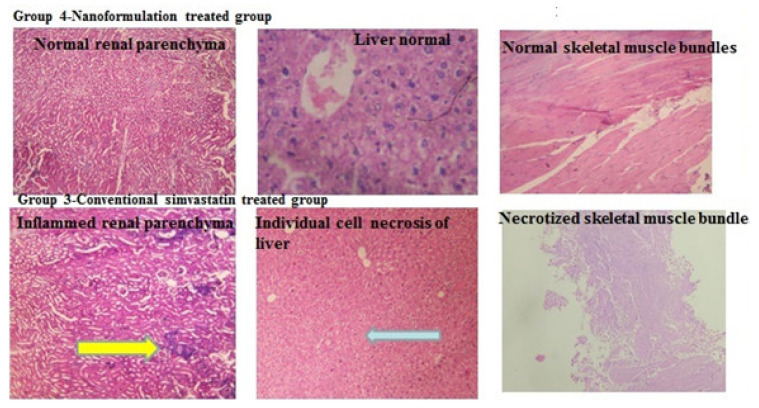
Comparative histopathology photographs of groups 3 and 4 representing reduced toxicity of group 4 (formulation treated).

In the control/HFD group of mice, the incidence of fiber necrosis was not observed (Figure 9. In the standard simvastatin-treated (group 3), all aspects of induced muscle necrosis were remarkably similar in all three mice. Most of the organs and muscles sampled from the hind limb except the soleus were affected by necrosis. Muscle necrosis was segmental, affected individual fibers, and characterized by loss of cytoplasmic structure, vacuolation, and little or no inflammatory infiltrate. In the nanoformulation F40-treated (group 4), muscle necrosis or negligible necrosis was not detected (Figure 10).

#### 2.6.2. Fiber Typing and Necrosis in Standard Simvastatin and Nanoformulation F40

The muscle fiber type of mammals is determined by the particular myosin heavy chain (MHC) isoform expressed. The limb muscles of the adult mice express one slow and three fast MHC isoforms [7]. Most fibers normally express only one isoform and are referred to as pure, although fibers are present that contain more than one [8]. The pure and mixed fiber types constitute a continuum from the slowest twitch type I fibers to the fastest twitch type IIB: I ↔ IC ↔ IIC ↔ IIA ↔ IIAD ↔ IID ↔ IIDB ↔ IIB. Statins cause type II muscle fiber degeneration particularly type II B is more sensitive than type I. Results showed that several muscles were totally or relatively saved in both the control and nanoformulation F40-treated groups. In the case of the standard simvastatin-treated group, the soleus muscle was insensitive to statin-induced necrosis. The soleus muscle consisted predominantly of type I fibers and a smaller proportion of type II A and a few II C fibers with no type II D or II B fibers. For type I and type II, fibers clearly showed that in muscles containing mixtures of these fibers, when early necrosis was present, then type I fibers were spared. Even when a substantial proportion of the type II fibers were necrotic, the type I fibers retained their normal histological appearance (Figure 10). This was consistent for the muscles which contained type I oxidative fibers biceps femoris. In fact muscle fiber, in which glycolysis is the major process, size is closely related to their metabolic characteristics; the lower the oxidative activity the greater the diameter of the fiber, with the largest fibers being type IIB. In this study, some muscles showed acute changes which contain type IIB fibers. Myosin ATPase staining confirms severe necrosis as observed in biceps brachii, vastus medialis.

### 2.7. Hemolysis Assay for Biocompatibility

This study showed that all the tested concentrations of formulation F40 neither showed hemolytic activity nor thrombus formation, making it a biocompatible systemic application. In addition to this, the group treated with SLS (positive control) showed 100% hemolysis marked by complete lysis of the red blood cells (RBCs). The PBS (negative control), drug, and nanoformulation F40 groups did not show any hemolysis or toxicity to the RBCs, revealing its possible biocompatibility. Hemolytic activity was further confirmed on a blood agar plate.

## 3. Discussion

Simvastatin is a medication with a significant problem of substantial first-pass metabolism, which results in a very low bioavailability of only 5%. The use of innovative medicine delivery techniques can boost this [9]. Presently, statin medications are typically taken orally by patients. In fact, this is the only method of taking statins that has received FDA approval. In addition to difficulties with poor absorption, which has driven novel statin formulations and different dose forms of statin administration, statins also carry a small but real risk of negative side effects [1,9].

In general, simvastatin is a P-gp inhibitor and due to this property, in the present study, the absorption of standard simvastatin was initially high, and it was decreased till the end of the experiment due to the efflux mechanism [10]. However, an increased absorption of the drug from nanoformulation F40 was due to its nano size, the presence of tween 80 as a surfactant in the formulation, and the encapsulating agent chitosan, which synergistically inhibits the P-gp efflux mechanism [11,12]. The integrity of tight junctions (TJs) can be altered by its opening through chitosan when delivered in the form of nanoparticles, rendering enhanced paracellular permeability in vivo. The gastrointestinal transit time is also altered by nanoformulation F40 due to its mucoadhesive property influencing its absorption and carrier-mediated uptake due to a decrease in the gut degradation of simvastatin. CYP3A44 is the most abundant cytochrome P450 enzyme within the intestinal enterocytes, responsible for metabolic elimination of simvastatin, causing retarded bioavailability. Nanoformulation F40 with encapsulation of the drug by chitosan demonstrated decreased gut metabolic elimination of simvastatin, thereby increasing oral bioavailability. As mentioned earlier, nanoformulation F40, due to the presence of chitosan, can adhere to epithelial surfaces, which in turn causes transient opening of tight junction (TJs) between adjacent cells. Transmembrane proteins and claudins regulate the specificity of tight junction permeability. Literature suggested that transmembrane protein CLDN4 plays a key role in chitosan-mediated reversible epithelial TJ opening [13]. The increased use of Polysorbate 80 for lipophilic drug candidates such as simvastatin which are P-gp substrates is needed [14,15]. Nanoformulation F40 demonstrated lower protein binding as compared to standard simvastatin due to the encapsulation by chitosan. This indicated more availability of the free drug to elicit the pharmacological action. As simvastatin is a narrow therapeutic index drug either higher protein binding or greater free drug concentration would be detrimental as it precipitates toxicity. The present study adopted one of the most effective strategies of encapsulation of simvastatin for better bioavailability and prolongation of the circulation time without any toxicity. Nanoformulation F40 demonstrated successful encapsulation and hydrophilicity due to the use of chitosan, PVA, and Tween 80 in the formulation, which in turn imparted reduced protein binding [16]. In this study, the assay was conducted over a 2 h period, resulting in less drug concentration being released. This was compared to the bulk of the drug that was available at this time with the unencapsulated drug. Therefore, the mechanism of protein binding for nanoformulation F40 may be considered as a function of the affinity of the polymeric nanoparticles for plasma proteins as well as the concentration of the drug available for binding which, at a specific time, is decreased by encapsulation [17]. The binding of albumins to the hydrophilic chitosan surface exerts the long residency of nanoformulation F40, thereby offering longer half-lives as evident from the PK studies. The chitosan coating in nanoformulation F40 also minimized opsonization, which will eventually prolong the systemic circulation of the nanoparticles. More specifically, in our study, the protein interaction was reduced at the highly curved surfaces of the nanoparticle. The spherical NPs have a higher association in the cell as compared to rod-shaped NPs, which is among the reasons in this study for almost 77% protein binding of nanoformulation F40 [18,19]. This study, however, focused on the effect of surface-coated simvastatin on in vitro protein binding to validate biodistribution nanoformulation F40 after in vivo evaluation. It is generally accepted that simvastatin and simvastatin acid in the liver are metabolized via CYP3A4. The results clearly demonstrated marked detraction of the activity in liver of the formulation F40-treated group compared to the standard simvastatin group, which was consistent with the decrease in simvastatin metabolism. This result suggested that the decreased activity and expression of hepatic CYP3A4 in the formulation F40 group were the main contributors to the reduced systemic clearance of simvastatin and simvastatin acid which increases the half-life (pharmacokinetic parameters). Nanoformulation F40 demonstrated the downregulation of intestinal and liver CYP3A4 mRNA levels, which were responsible for systematic metabolism and first-pass metabolism of simvastatin, respectively. As the simvastatin and simvastatin acid were encapsulated by chitosan, this left no scope for metabolism by intestinal CYP3A4, leading to suppression of CYP3A4. All the results support the conclusion that the downregulation of both CYP3A4 activity and expression decreases the hepatic metabolism of simvastatin in formulation F40, thus leading to long exposures of simvastatin and simvastatin acid.

The metabolic pathway of nanoformulation F40 was observed to be increased more with the activity of PPAR-γ than the standard simvastatin group, which is contradictory to the previously observed downregulation of PPAR-γ expression in hypercholesterolemic animals [20]. There was also a significant increase in the expression of PPAR-γ mRNA by nanoformulation F40 compared to the control and standard groups as evident from the qRT-PCR profiling. This study confirmed that simvastatin in formulation F40 can also improve the downregulated PPAR-γ mRNA expressions in the liver indirectly through the induction of SREBP-2. The study findings revealed a combination effect of chitosan and simvastatin, mechanizing through different pathways to synergize the cholesterol depletion effect. SREBP-2 mRNA expression was increased in the liver of standard simvastatin mice compared to the control and formulation F40 groups and this was accompanied by a significant increase in the mRNA expression of LDLr and HMG-CoAR, two SREBP-2 target genes. The cholesterol depletion effect observed due to nanoformulation F40 induces proteolytic activation of the SREBP family [21], cascade of which causes induction of PPAR-γ levels. This increase in the PPAR-γ transcriptional activity is expected to cause the expression of several genes responsible for triglyceride clearance [22,23]. Hence, this mode of interaction between transcription factors controlling different lipid pathways may provide somewhat unexpected/LDL-lowering effects. At this point, some well-designed studies need to be initiated to prove this.

The present study demonstrated a significant increase in the excretion of cholesterol (4-fold) and bile acids in feces. For the nanoformulation-treated group, the cholesterol concentration was found to be 0.49 ± 0.005 mg/day/animal, which was higher when compared to the standard-treated group (0.13 ± 0.008 mg/day/animal). Nanoformulation F40 feeding has been reported to increase the fecal excretion of cholesterol, its non-polar derivatives and bile acids. The present study demonstrated two different mechanistic pathways for serum cholesterol reduction, the SREPB-2 pathway being followed by simvastatin and the PPAR-γ pathway being followed by chitosan. The higher efficacy of nanoformulation F40 in terms of fecal elimination of cholesterol and bile acids was due to the synergistic effect produced by simvastatin and chitosan. Chitosan is primarily responsible for the above effect as it upregulates fecal excretion upon hypercholesterolemia. Previous studies in animal models [24] as well as in humans [13] also supported the present findings.

In this study, using a low-molecular-weight, high-DD chitosan, nanoparticle size added value in high binding and excretion of lipids in feces. This study also indicated that chitosan lowered the plasma total cholesterol level by enhancement of the hepatic LDL receptor mRNA and chitosan in formulation F40 might have the potential to increase the excretion of fecal bile acids. The use of chitosan, PVA, and surfactant Tween 80 in the nanoformulation is responsible for the increased dissolution of simvastatin and influx against P-gp activity. The previous in vitro studies showed that the lactone ring in formulation F40 showed maximum stability in gastric pH, and no degradation of the lactone in 24 h was observed [25,26].

However, in the present research, significantly higher MRT values of metabolite SSA from nanoformulation F40 in comparison to the standard drug are responsible for the prolonged residence of metabolite in rabbits. Thus, it might be expected that the enhanced residence would have a positive effect on the efficacy of the active metabolite. It appeared that nanoformulation F40 delivered SS in a more sustained fashion, providing smoother plasma concentration profiles and lower maximum plasma concentrations compared with those of standard simvastatin. Since increased peak concentrations of SS are related to the incidence of adverse events, the obtained smooth plasma concentration coupled with a lower C_max_ and a higher AUC values could potentially reduce the incidence of such toxic events and could sustain the efficacy of SS at the same time. This nano-controlled drug delivery system resulted in a lower plasma concentration, but it provides a constant pharmacological availability of the drug which might reduce toxic side effects [27]. Mucoadhesive functionalities of chitosan and PVA in the formulation amplify the potentiality in terms of drug transport across the intestinal barrier to produce its hypolipidemic action. The studied nanoformulation F40 proved to be an optimized and balanced delivery modality for simvastatin in terms of a persistent, controlled PK profile and reduced adverse effects as well as safety. Compared to the conventional drug, nanoformulation F40 indicated reduced muscle toxicity as evidenced by histology. Chitosan encapsulation prevents the opsonization process and improves the biocompatibility of nanoformulation F40, which was further evidenced by the results of biocompatibility studies.

## 4. Materials and Methods

### 4.1. Materials

Nanoformulation F40 was designed, formulated, and optimized in our laboratory and the results are published [6]. Briefly, the nanoparticles of simvastatin were prepared by a solvent evaporation method using the different mass ratio of the drug to chitosan solution (2% acetic acid). The 0.5% polyvinyl alcohol and 0.2% tween 80 were used as stabilizers and surfactants, respectively. By subjecting to various steps of methodology viz., magnetic stirring, homogenization, centrifugation, and freeze-drying, nanoparticles were prepared. Nanoparticles exhibited a narrow size distribution, a higher positive zeta potential, and greater encapsulation efficiency with amorphous conversion. The modified physicochemical properties of simvastatin in the nanoformulation were attributed to the decrease in LDL, TG, and total cholesterol and increase in HDL with a several-fold reduced dose of simvastatin when compared to pure drug. The present study deals with the PK functionalities to prove the efficiency and efficacy of nanoformulation F40 CS-SS. The drug simvastatin used to formulate F40 was a gift from Biocon Pvt. Ltd., India. All other chemicals, polymers, and solvents utilized in the present work were of analytical grade and have been purchased from Sigma Aldrich, Germany.

### 4.2. Methodology

#### 4.2.1. The Everted Sac Technique: In Vitro Absorption Study

Female albino mice (24–30 g) were used for this study and were obtained from King Institute, Chennai. The animals were kept in a controlled environment of 25 °C with a 12 h light/12 h dark cycle and all protocols and procedures used were approved by the IAEC PRIST University, Thanjavur (Project 1 Pon/Phar1/2015). The mice were fasted overnight before experimentation and had access to water *ad libitum*. The experiment was performed as per the reported method [28,29]. Briefly, 5 cm of the jejunum of the intestine was maintained with an ice-cold physiological solution and everted. Then, it was filled with 600 µL Krebs solution and sealed. It was then transferred into the incubation flask containing test samples in 25 mL oxygenated media at 37 °C. The sampling was performed at different time intervals and evaluated.

#### 4.2.2. In Vitro Plasma Protein Binding: Distribution Studies

The in vitro bovine serum albumin binding test was performed by the most popular method of equilibrium dialysis with the use of an activated 10–12 kDa molecular cut-off dialysis membrane [29]. It was filled with standard BSA solution and required a concentration of samples (pure simvastatin and nanoformulation F40) and the volume was maintained up to 4 mL. The membrane bags were immersed in conical flasks containing phosphate buffer solution and were shaken gently at 37 ± 0.5 °C for approximately 6 h in a shaking incubator. After shaking, the absorbance of free drug in the buffer (outside the membrane bags) was measured at 238 nm using the UV–VIS spectrophotometer, and the concentrations of the bound and unbound drugs were determined using a standard curve. The pure drug was taken as control.

$$\%\;Bound=\frac{\left(1-Concentration\;of\;free\;drug\;in\;test\;sample\right)}{Concentration\;of\;free\;drug\;in\;control}\times 100$$

In vitro protein binding of nanoformulation F40 was calculated using the above equation [30]. Mice plasma and human plasma were used for the assay with the approval of the ethical committee.

#### 4.2.3. qRT-PCR: Metabolic Pathway Determination

##### Separation of m-RNA

For separation of m-RNA, the mice were anesthetized for collecting the blood and other organs were dissected and immediately frozen in liquid nitrogen.

##### RNA Isolation by Trizol Method

For direct lysis of the cells, 1 mL of TRI reagent was added per 1000 mg of the tissue sample. The processed samples were centrifuged at 12,000× *g* for 15 min at 2–8 °C, which separated the mixture into three phases: a red organic phase (containing protein), an interphase (containing DNA), and a colorless upper aqueous phase (containing RNA). The upper aqueous phase was processed further and allowed to stand for 5–10 min at room temperature and centrifuged at 12,000× *g* for 10 min at 4 °C. The supernatant was carefully aspirated off the tube, leaving behind the precipitated RNA pellet on the sides of the tube, which was washed by adding a minimum of 1 mL of 75% ethanol per 1 mL of TRI reagent. The sample was then vortexed and then centrifuged at 7500× *g* for 5 min at 2–8 °C. The RNA pellet was air-dried and suspended in 20–30 μL of RNase-free water [31].

##### qRT-PCR (Quantitative Reverse Transcriptase Polymerase Chain Reaction) for CYP3A4 Microenzyme Analysis

qRT-PCR was used to measure mRNA levels of CYP3A4 in the liver and intestine by quantitative RT-PCR analysis using a cDNA input converted from 2 μg of total RNA. Primer sequences of mice mRNA for CYP3A4 are Forward 5′-CAGGAGGAAATTGATGCAGTTTT-3′; Reverse 5′ GTCAAGATACTCCATCTGTAGCACAGT-3′. After denaturing at 95 °C for 2 min, the amplification was obtained by 40 cycles of 95 °C for 5 s and 60 °C for 30 s. Melting curves were obtained to investigate the specificity of the PCR reaction. For normalization of the gene levels, β-actin (primer sequence—Forward 5′-CAGTCGGTTGGAGCGAGCAT-3′ Reverse 5′-GGACTTCCTGTAACAACGCATCT-3′) was used to correct minor variations in the input RNA amount or inefficiencies of the reverse transcription. The relative quantification (RQ) of each gene expression was calculated according to the comparative Ct method using the formula: RQ = 2^−ΔCt^ [31].

##### Determination of Metabolic Pathway of Simvastatin and Chitosan in the Standard Simvastatin and Formulation F40-Treated Groups—qRT-PCR

After extraction of RNA using TRI reagent as explained above, the mRNA expression for PPAR-γ, and SREPB2 was carried out in the liver using an RT-PCR kit from PROMEGA by using a standard protocol. The PCR reaction was performed in the thermal cycle with RT reaction at 40 °C for 30 min, initial PCR activation at 94 °C for 2 min, followed by 35 cycles of 94 °C (denaturation) for the 30 s, 58 °C (annealing) for 30 s, and 72 °C (extension) for 1 min. Finally, the reaction mixture was incubated at 72 °C for 10 min to extend any incomplete single strands. Optimal oligonucleotide primer pairs for RT-PCR were selected with the aid of the software Gene Runner. The primer sequence (5′ to 3′) for mice gene coding (+) strand was: PPARγ (Sequence ID: NM_001145366.1); Forward: 5′-GCCCTTTGGTGACTTTATGG-3′; Reverse: 5′CAGCAGGTTGTCTTGGATGT-3′; β-actin (Sequence ID: NM_031144); Forward: 5′-CACCCGCGAGTACAACCTTC-3′; Reverse: 5′-CCCATACCCACCATCACACC-3′; SREBP2; Forward primer 5′ to 3′ CCAAAGAAGGAGAGAGGCGG; Reverse primer 5′ to 3′ CGCCAGACTTGTGCATCTTG. Melt curve analyses were obtained with each series to confirm the specificity of the primers and products were amplified by heating the amplified products from 658 °C to 958 °C at 0.58 °C steps for 5 s. Relative quantification of mRNA expression was analyzed by the 2^−ΔCt^ method [31].

#### 4.2.4. Fecal Matter Evaluation—In Vitro Excretion

For the fecal matter evaluation, mice were individually housed in polypropylene cages at a controlled room temperature at 25 °C, under a 12 h light/12 h dark cycle and with free access to food and water. The mice were randomly divided into four experimental groups (n = 6). Group 1 was fed a standard laboratory diet (CD). Group 2 was fed a cholesterol-rich diet (HFD). Groups 3 and 4 received HFD with standard drug and formulation F40 (10 mg/kg). The duration of the treatment was 16 weeks. Each treatment group received a standard cholesterol diet daily orally in the morning throughout 16 weeks to induce hyperlipidemia except the control treatment group. A high-cholesterol diet was prepared by mixing cholesterol 2%, sodium cholate 1%, and coconut oil 2% with animal food. Throughout this study, various parameters were monitored and measured which includes bodyweight, food intake, fecal dry weight, total cholesterol concentration in feces (mg/day/animal), total bile acids in feces (mg/day/animal), simvastatin concentration in ng/mL, and simvastatin metabolite in ng/mL.

##### Determination of Fecal Cholesterol and Bile Acid Contents

Fecal total sterol and total fecal bile acids were extracted from dry feces and quantified enzymatically according to the procedures provided in the assay kit purchased from Sigma Aldrich. Neutral sterols (NSs) in feces were determined as previously described [32], with some modifications. Briefly, after extraction of sterol, it was incubated with 5.0 mL alcoholic KOH solution and 10 mL of petroleum ether. Then, 5.0 mL of distilled water was added. An aliquot (0.5–2.0 mL) of the supernatant was taken into test tubes and petroleum ether was evaporated under nitrogen. To each of the samples, 3.0 mL of glacial acetic acid, and 0.1 mL distilled water were added and then 2.0 mL of the FeC1_3_-H_2_SO_4_ reagent was added. The intensity of immense purple color formed was measured in a Shimadzu-UV spectrophotometer at 560 nm. The bile acids and total cholesterol were determined by an enzymatic method [15]. Working Reagent was prepared by mixing an appropriate quantity of Assay Buffer, NAD, Probe, Enzyme A, and Enzyme B. The specified amount of working Reagent was added to Internal Standard (sodium cholate) and sample wells, and Blank Reagent was added to the sample blank wells. The plate was taped to mix and incubated for 20 min in the dark. The fluorescence intensity at 585 nm was read. The bile acid concentration of a sample was calculated using a formula. The extract was mixed with mobile phase (chloroform-isopropyl alcohol-ammonium hydroxide in the ratio 20:25:1 for bile acid separation; benzene-diethyl ether in the ratio 85:5 for sterol separation) and HPTLC analysis was carried out

#### 4.2.5. In Vivo Pharmacokinetic Study

Albino rabbits of either sex weighing 1.5 to 3.0 kg were used for the estimation of pharmacokinetic parameters of nanoformulation F40 and standard simvastatin. The animal study protocol was approved by IAEC in presence of the CPCSEA nominee with Approval no. Project 1 Pon/Phar/2015 at PRIST University, Thanjavur. Pharmacokinetic studies were carried out based on a single dose complete cross-over method in twelve healthy albino rabbits. The rabbits were weighed and randomly divided into two groups, standard and test with six animals in each group. The data were acquired and calculated on Shimadzu controlled using a software analyst. The pharmacokinetic parameters for pure simvastatin and nanoparticles following oral administration were determined from plasma concentration data. The area under the concentration–time curve *AUC*_(0–*t*)_ was estimated according to the trapezoidal rule. The area under the curve extrapolated to infinity *AUC*_(0–∞)_ was calculated by formula.

$$AUC_{\left(0-\infty \right)}=\frac{AUC_{\left(0-t\right)}+C_{last}}{K_{e}}$$

where *C_last_* and *K_e_* are the last measurable concentration and the elimination rate constant, respectively [16]. The elimination rate constant, half-life, mean residence time, and relative bioavailability were calculated using the below formula.

$$K_{e}=-2.303\times Slope$$

$$t_{1/2}=\frac{0.693}{K_{e}}$$

$$MRT=1.44\times t_{1/2}$$

$$Fr\;\%=\frac{AUC_{\left(0-\infty \right)}\;Formulation\;F40_{\;}}{AUC_{\left(0-\infty \right)}Reference}\times 100$$

#### 4.2.6. Histopathological Analysis (Toxicity)

The animals were sacrificed as per CPCSEA protocol. The heart, kidney, liver, stomach, brain, and a range of muscle tissues were sampled for necropsy and histology. The muscle tissues sampled were biceps femoris, soleus, tibialis cranialis, vastus medialis from the left hind limb; biceps brachii from the left forelimb. Tissues were fixed in buffered 10% formalin, processed to wax blocks, and then sectioned and stained with hematoxylin and eosin for examination by light microscopy. During the histopathological examination under microscopy, the presence or absence of necrosis was observed for all four groups.

#### 4.2.7. Biocompatibility Analysis: Hemolytic Activity on Human Blood Agar Plate

In 95 mL of sterile nutrient agar placed in Petri dishes, 5.0 mL of human blood was added aseptically and allowed for solidification. Then, wells were cut into the agar plate using a corkscrew borer (8 mm diameter) and loaded with 50 μL (1 mg/mL) of samples. The plates were observed for hemolysis after overnight incubation at room temperature [33].

### 4.3. Statistical Analysis

All the data were presented as the mean ± SD and analyzed by the statistical software package GraphPad Prism 5 version (GraphPad Software, San Diego, CA, USA). The statistical analysis included a one-way analysis of variance (ANOVA) followed by the Tukey post hoc test. The difference between the two parameters was considered statistically significant for *p* < 0.05.

## 5. Conclusions

The present study represents an important contribution since it demonstrated a promising pharmacokinetic profile and ideal safety of nanoformulation F40 as a unique synergistic hypolipidemic modality. Pure simvastatin has less absorption due to dissolution-limited absorption, the P-gp efflux mechanism, gut/liver metabolism, being highly protein bound, and less elimination at half-life. Although its AUC is not particularly high, it produces toxicity in tissues through liver and muscle. In the present study, the use of a hydrophilic polymer in the nanoformulation improves the wettability of the drug and dissolution. Utilization of surfactant Tween 80 modulates the P-gp efflux mechanism, allowing penetration of more drug and a higher distribution volume. This increase in absorption and protective effect of chitosan decrease gut/liver metabolism. Therefore, a higher percentage of the drug is distributed in body fluids entering plasma. When a positive surface charge reduces protein binding, an increase in the volume of distribution (V_d_, 378.90 ± 112.31 and 404 ± 134.98 for simvastatin and its acid in nanoformulation, respectively) was noticed. In addition, in plasma, controlled metabolism of simvastatin to simvastatin acid occurs. This all causes a higher T_max_ of 10.00 ± 2.78 (simvastatin) and 14.56 ± 2.19 (simvastatin acid), and a lower C_max_. Drug elimination by biliary and feces was also altered due to increased drug transporter P-gp activity present in the intestine, liver, and kidney exerted by Tween-80. This causes decreases in the V area (volume at the terminal phase of elimination), clearance, and an increase in half-life (12.29 ± 4.57 for simvastatin and 16.87 ± 3.91 for simvastatin acid) on the whole representing a higher AUC and relative bioavailability. The reduced dose subsequently decreased muscle-related toxicity, which was confirmed by histopathological analysis. Finally, it can be concluded that this hypolipidemic action of the F40 CS-SS nanoformulation may be due to inhibition of the absorption of dietary cholesterol by the biliary secretion of cholesterol and cholesterol excretion in the feces caused by chitosan and its reduced production by the liver induced by simvastatin. Thus, the multifunctional properties of chitosan not only modify drug properties but also importantly synergize the hypolipidemic activity of simvastatin. Thus, the promising in vitro and In vivo results obtained in this research suggest pathways for future research.

## Figures and Tables

**Table 1:** The concentration of simvastatin in the mucosal side of everted intestine for the pure sample and CS-SS nanoformulation.

Time in Min	Concentration μg/mL in Mucosal Side	
	Standard	CS-SS Nanoformulation
0	25 ± 1.21	27 ± 0.90
15	20 ± 1.01	26 ± 0.70
30	22 ± 1.90	26 ± 0.90
45	19 ± 1.30	22 ± 0.56
60	20 ± 1.00	18 ± 0.43
75	23 ± 2.10	16 ± 0.41

Results are expressed as the mean ± SD.

**Table 2:** Percentage of plasma protein binding of pure simvastatin and CS-SS nanoformulation.

Binding Type	Pure Simvastatin (%)	CS-SS Nanoformulation (%)
Binding with bovine serum albumin	95.00 ± 3.11	77.02 ± 4.58
Binding with human plasma	95.70 ± 1.00	81.80 ± 1.90
Binding with mice plasma	96.00 ± 0.73	83.10 ± 2.22

Results are expressed as the mean ± SD.

**Table 3:** Results depicting the excretion parameters of CS-SS nanoformulation.

Parameters	Time	Control Group	HFD Group	Simvastatin-Treated Group	CS-SS Nanoformulation-Treated Group
Bodyweight in mg	Initial	24.50 ± 2.01	24.00 ± 2.30	24.01 ± 3.30	24.50 ± 3.10
	8 weeks	26.30 ± 1.80	30.70 ± 3.22	30.20 ± 1.90	29.04 ± 3.04
	16 weeks	28.90 ± 0.90	34.00 ± 5.00	32.90 ± 2.40	29.20 ± 2.10
Food intake in mg	Initial	1.50 ± 0.20	1.50 ± 0.20	1.50 ± 0.21	1.50 ± 0.20
	8 weeks	1.62 ± 0.41	1.89 ± 0.67	1.52 ± 0.30	1.48 ± 0.11
	16 weeks	1.69 ± 0.54	1.96 ± 0.34	1.52 ± 0.81	1.46 ± 0.13
Fecal dry weight	Initial	0.12 ± 0.003	0.10 ± 0.004	0.12 ± 0.003	0.10 ± 0.002
	8 weeks	0.13 ± 0.007	0.18 ± 0.001	0.13 ± 0.00	0.32 ± 0.002
	16 weeks	0.12 ± 0.007	0.27 ± 0.020	0.13 ± 0.008	0.49 ± 0.005
Total cholesterol concentration in feces mg/day/animal	Initial	Traces	Traces	Traces	Traces
	8 weeks	Traces	Traces	Traces	2.8 ± 1.2
	16 weeks	Traces	Traces	Traces	3.4 ± 0.9
Total bile acids in feces mg/day/animal	Initial	Traces	Traces	Traces	Traces
	8 weeks	Traces	Traces	Traces	5.9 ± 0.87
	16 weeks	Traces	Traces	Traces	8.2 ± 1.43
Simvastatin concentration in ng/mL	16 weeks	NA	NA	18.98 ± 0.20	NF
Simvastatin metabolite in ng/mL	16 weeks	NA	NA	23.12 ± 1.3	Traces

Results are expressed as the mean ± SD for *n* = 6.

**Table 4:** Pharmacokinetic parameters of simvastatin and simvastatin acid (metabolite) in pure drug and nanoformulation.

Parameters	Standard Simvastatin *	Standard Simvastatin Active Metabolite *	Simvastatin in CS-SS Nanoformulation *	Simvastatin Active Metabolite in CS-SS Nanoformulation *	*p* Value
C_max_ (ng/mL)	21.12 ± 7.24	19.42 ± 6.90	4.33 ± 1.70	3.98 ± 1.60	0.005 *
T_max_ (h)	04.72 ± 1.20	5.00 ± 0.31	10.00 ± 2.78	14.56 ± 2.19	0.001 *
t_½_ (h)	1.343 ± 0.689	4.20 ± 2.20	12.29 ± 4.57	16.87 ± 3.91	0.001 *
K_E_ (h–1)	0.57 ± 0.140	0.420 ± 0.060	0.0445 ± 0.008	0.0195 ± 0.0147	0.001 *
Vd (L/Kg)	163.20 ± 79.00	222.15 ± 69.35	378.90 ± 112.32	404.00 ± 134.98	0.050 *
AUC last (nghr/mL)	35.09 ± 12.23	42.72 ± 10.00	52.17 ± 9.86	73.11 ± 12.56	0.016 *
AUC(0–∞) (nghr/mL)	36.38 ± 10.90	51.11 ± 14.00	54.35 ± 8.31	73.98 ± 11.90	0.024 *
Cl (mL/min/kg)	246.88 ± 121.62	378.30 ± 96.22	135.78 ± 77.06	180.34 ± 90.00	0.049 *
MRT	2.00 ± 1.10	9.00 ± 2.30	14.98 ± 3.40	17.85 ± 2.87	0.001 *
Fr (%)	100.00 ± 0.0	100.00 ± 0.0	154.46 ± 23.41	209.66 ± 31.53	0.001 *

Notes: All values are the means ± SD (n = 3). * indicates the significance and the *p* values found to be significant for the C_max_, T_max_, t_1/2_, K_E_, V_d_, AUC last AUC(0–∞), Cl, and MRT at *p* < 0.05. Details are given in the Supplementary Materials.

## Data Availability

The data presented in this study are available on request from the corresponding author. The data are not publicly available due to university regulations.

## Supplementary Materials

The following supporting information can be downloaded at: https://www.mdpi.com/article/10.3390/ph16030380/s1.
