# Supplementary material for: A Study on Pharmacokinetic Functionalities and Safety Margins of an Optimized Simvastatin Nanoformulation
Source: Pharmaceuticals (Basel). 2023 Mar 1;16(3):380. doi: 10.3390/ph16030380 (PMC10056947; doi:10.3390/ph16030380)

SUPPLEMENTARY FILE

Table A

Pharmacokinetic parameters of simvastatin and simvastatin active metabolite.

Parameters	Simvastatin	Simvastatin Active Metabolite	Student Independent t Test	P Value
	Mean ± SD			
C_{max} (ng/ml)	21.12 ± 7.24	19.42 ± 6.90	0.294	0.783 NS
T_{max} (hr)	4.72 ± 1.20	5.00 ± 0.31	0.391	0.716 NS
$t_{½}$ (hr)	1.343 ± 0.689	4.20 ± 2.20	2.149	0.098 NS
K_E (hr^{-1})	0.516 ± 0.140	0.420 ± 0.060	1.092	0.336 NS
V_d (L/Kg)	163.20 ± 79.00	222.15 ± 69.35	0.971	0.386 NS
AUC_{last} (ng hr /ml)	35.09 ± 12.23	42.72 ± 10.00	0.837	0.450 NS
$AUC_{(0-\infty)}$ (ng hr /ml)	36.38 ± 10.90	51.11 ± 14.00	1.438	0.224 NS
Cl (ml/min/kg)	246.88 ± 121.62	378.30 ± 96.22	1.468	0.216 NS
MRT	2.00 ± 1.10	9.00 ± 2.30	4.756	0.009 **
Fr (%)	100.00 ± 0.0	100.00 ± 0.0	NA	NA

All values are mean ± SD (n = 3). P values were found to be Non-significant denoted as NS and the symbol * indicates significance at P < 0.05 and NA denotes Not Applicable

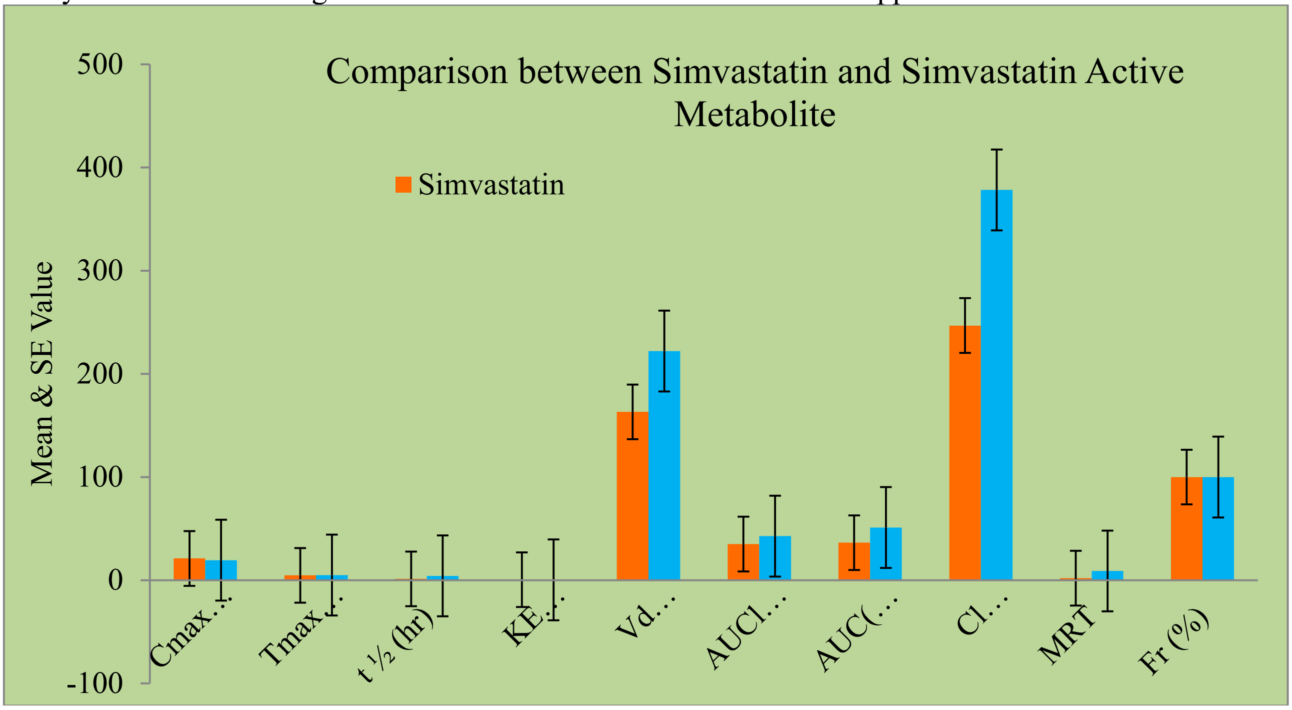

Table B

Pharmacokinetic parameters of simvastatin and simvastatin active metabolite in the formulation.

Parameters	Simvastatin in formulation	Simvastatin active metabolite in the formulation	Student Independent t Test	P Value
	Mean ± SD			
C_{max} (ng/ml)	4.33 ± 1.70	3.98 ± 1.60	0.260	0.808 NS
T_{max} (hr)	10.00 ± 2.78	14.56 ±2.19	2.232	0.089 NS
$t_{½}$ (hr)	12.29±4.57	16.87±3.91	1.319	0.258 NS
K_E (hr^{-1})	0.0445±0.008	0.0195±0.0147	2.580	0.061 NS
V_d (L/Kg)	378.90±112.32	404.00±134.98	0.247	0.817 NS
AUC_{last} (ng hr /ml)	52.17±9.86	73.11±12.56	2.271	0.086 NS
$AUC_{(0-\infty)}$ (ng hr /ml)	54.35±8.31	73.98±11.90	2.343	0.079 NS
Cl (ml/min/kg)	135.78±77.06	180.34±90.00	0.651	0.550 NS
MRT	14.98±3.40	17.85±2.87	1.117	0.326 NS
Fr (%)	154.46±23.41	209.66±31.53	2.434	0.072 NS

All values are mean ± SD (n = 3). P values were found to be Non-significant denoted as NS and the symbol * indicates significance at P < 0.05.

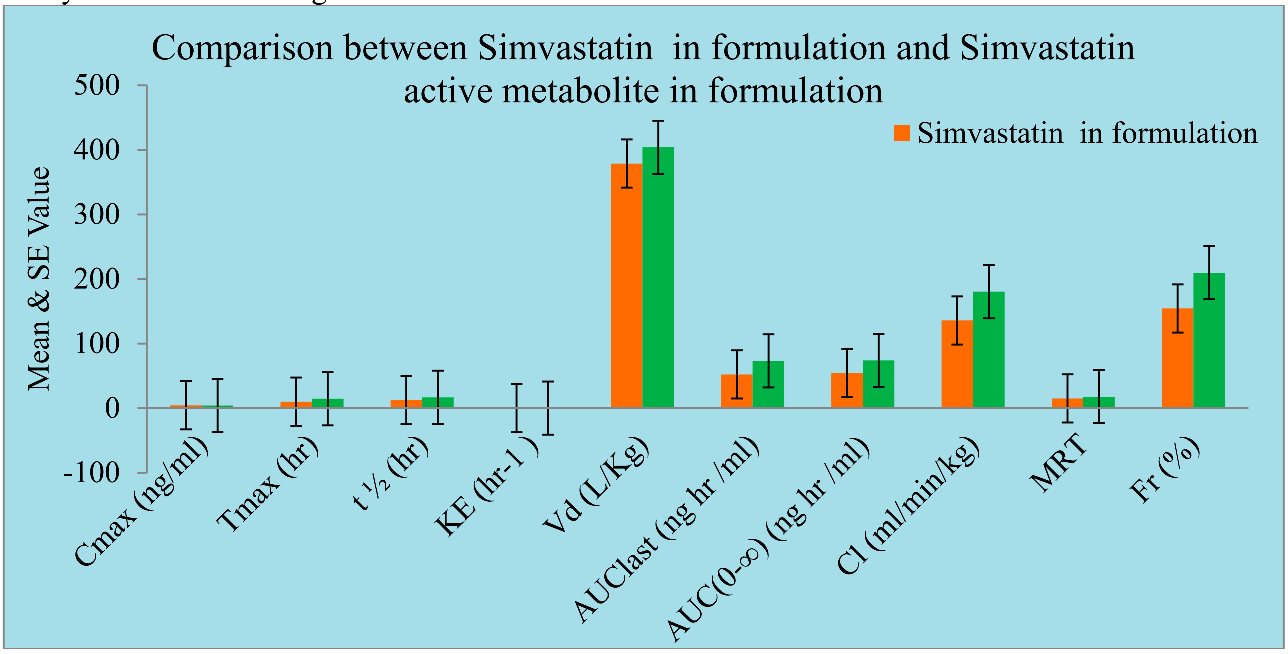

Table C

Pharmacokinetic parameters of simvastatin standard drug and simvastatin in the formulation.

**Parameters**	**Standard drug Simvastatin**	**Simvastatin in formulation**	**Student Independent t Test**	**P Value**
	**Mean ± SD**			
C_{max} (ng/ml)	21.12±7.24	4.33±1.70	3.91	0.017 *
T_{max} (hr)	4.72±1.20	10.00±2.78	3.020	0.039 *
$t_{½}$ (hr)	1.34±0.68	12.29±4.57	4.104	0.015 *
K_E (hr^{-1})	0.516±0.140	0.0445±0.008	5.824	0.004 **
V_d (L/Kg)	163.20±79.00	378.99±79.00	2.722	0.053 NS
AUC_{last} (ng hr /ml)	35.09±12.23	52.17±9.86	1.883	0.133 NS
$AUC_{(0-\infty)}$ (ng hr /ml)	36.38±10.90	54.35±8.31	2.271	0.086 NS
Cl (ml/min/kg)	246.88±121.62	135.78±77.06	1.337	0.252 NS
MRT	2.00±1.10	14.98±3.40	6.291	0.003 **
Fr (%)	100.00±0.00	154.46±23.41	4.029	0.016 *

All values are mean ± SD (n = 3). P values were found to be Non-significant denoted as NS and the symbol * indicates significance at $P < 0.05$.

Graphical representation related to Table 4 of the manuscript. Pharmacokinetic parameters (C_{max} and T_{max}) of simvastatin and simvastatin acid (metabolite) in pure drug and nanoformulation. P values are expressed in Table 4 of the manuscript.

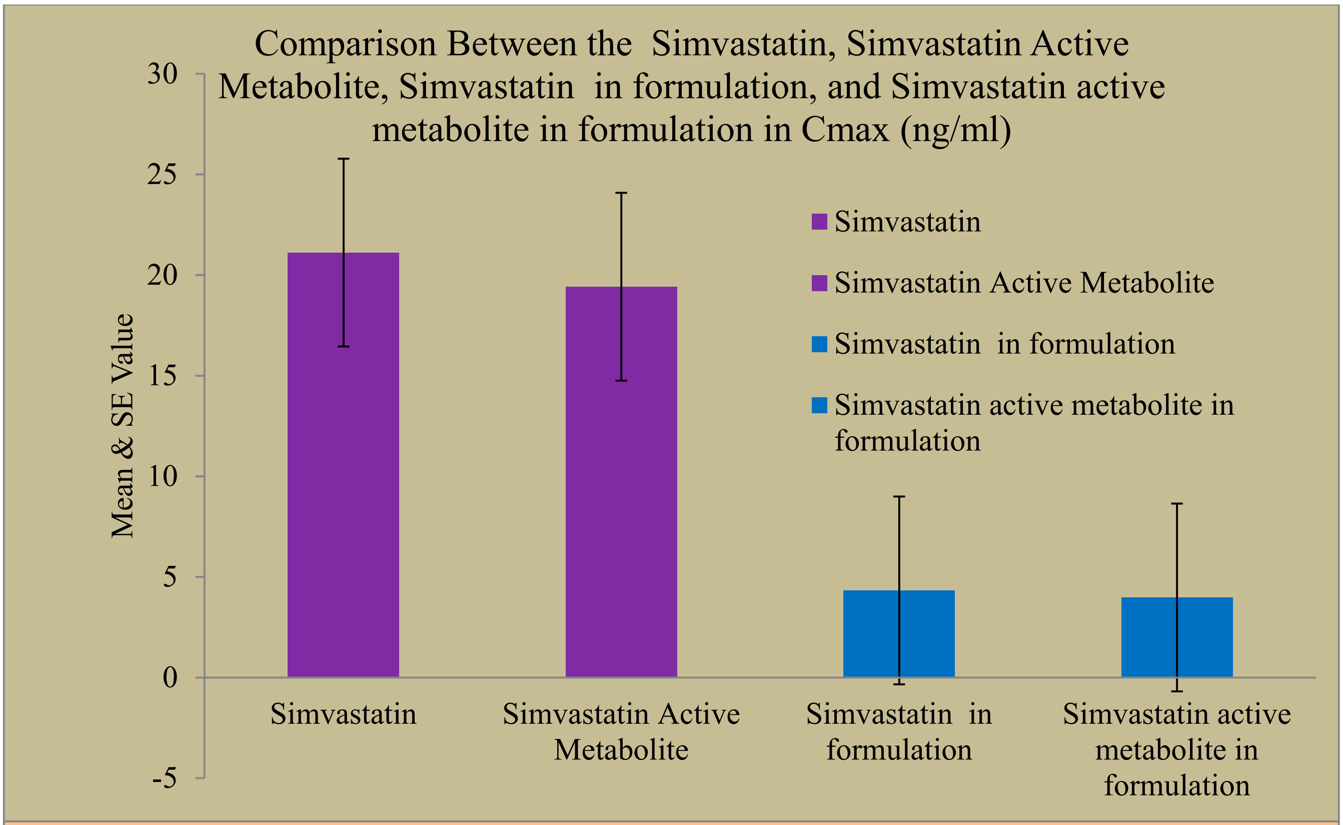

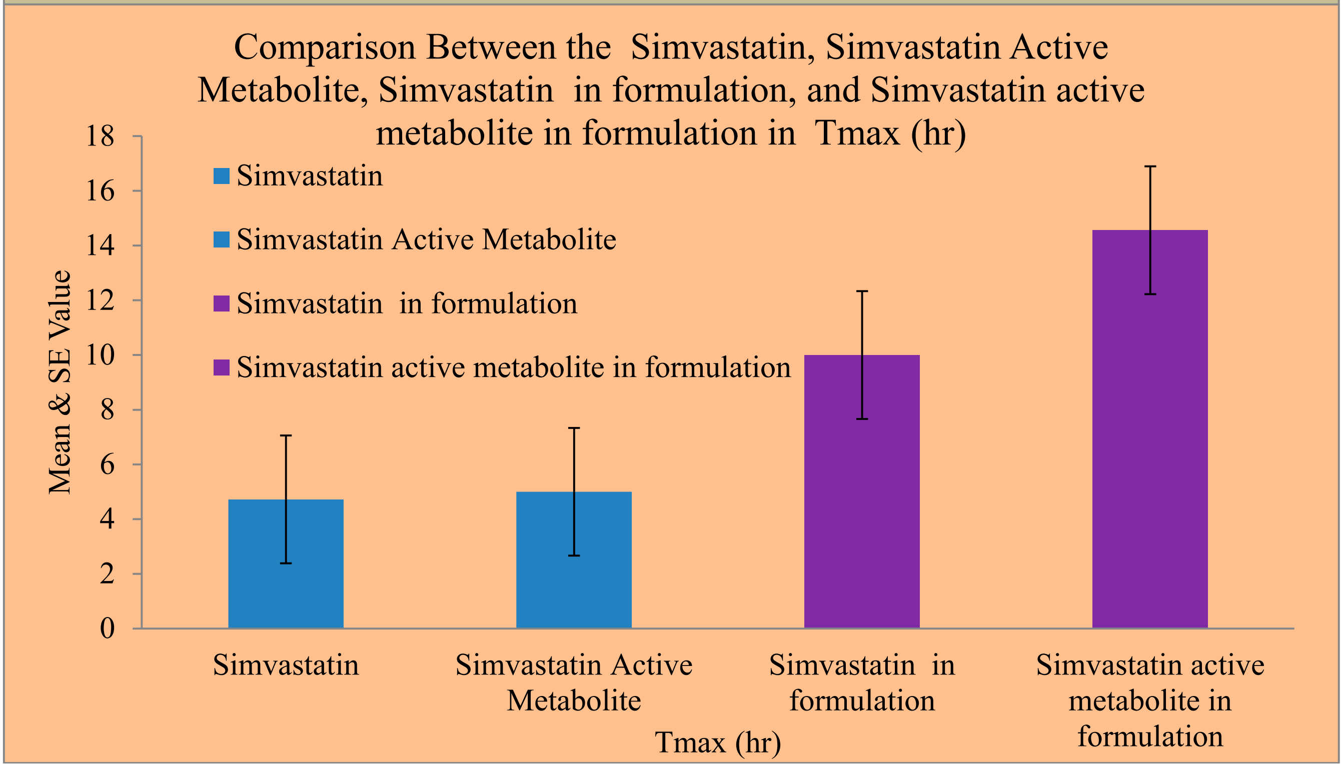

Supplement: Supplementary file 1 [file pharmaceuticals-16-00380-s001.zip › pharmaceuticals-2229871-supplementary.pdf]
